# Genome Sequencing and Phylogenetic Analysis of 39 Human Parainfluenza Virus Type 1 Strains Isolated from 1997–2010

**DOI:** 10.1371/journal.pone.0046048

**Published:** 2012-09-27

**Authors:** Eric T. Beck, Jie He, Martha I. Nelson, Michael E. Bose, Jiang Fan, Swati Kumar, Kelly J. Henrickson

**Affiliations:** 1 Midwest Respiratory Virus Program, Department of Pediatrics, Medical College of Wisconsin, Milwaukee, Wisconsin, United States of America; 2 Children's Research Institute, Children's Hospital of Wisconsin, Milwaukee, Wisconsin, United States of America; 3 Fogarty International Center, National Institutes of Health, Bethesda, Maryland, United States of America; University of Hong Kong, China

## Abstract

Thirty-nine human parainfluenza type 1 (HPIV-1) genomes were sequenced from samples collected in Milwaukee, Wisconsin from 1997–2010. Following sequencing, phylogenetic analyses of these sequences plus any publicly available HPIV-1 sequences (from GenBank) were performed. Phylogenetic analysis of the whole genomes, as well as individual genes, revealed that the current HPIV-1 viruses group into three different clades. Previous evolutionary studies of HPIV-1 in Milwaukee revealed that there were two genotypes of HPIV-1 co-circulating in 1991 (previously described as HPIV-1 genotypes C and D). The current study reveals that there are still two different HPIV-1 viruses co-circulating in Milwaukee; however, both groups of HPIV-1 viruses are derived from genotype C indicating that genotype D may no longer be in circulation in Milwaukee. Analyses of genetic diversity indicate that while most of the genome is under purifying selection some regions of the genome are more tolerant of mutation. In the 40 HPIV-1 genomes sequenced in this study, the nucleotide sequence of the L gene is the most conserved while the sequence of the P gene is the most variable. Over the entire protein coding region of the genome, 81 variable amino acid residues were observed and as with nucleotide diversity, the P protein seemed to be the most tolerant of mutation (and contains the greatest proportion of non-synonymous to synonymous substitutions) while the M protein appears to be the least tolerant of amino acid substitution.

## Introduction

Human parainfluenza virus type 1 (HPIV-1) causes upper and lower respiratory infections (URI, LRI) in infants, young children, and the elderly. The virus also causes more severe disease in immunocompromised patients and those with chronic medical conditions [1]–[3]. Children under 5 years old and particularly those children younger than 1 year are the most commonly hospitalized by HPIV-1 infection [4]. In the United States, the rate of HPIV-1-associated hospitalization among children younger than 5 years is estimated at 0.32–1.6 per 1000 children [1]. Estimates of annual hospitalizations in the U.S. due to HPIV-1 vary from 5,800 to 28,900 [1], [5].

HPIV-1 is one of four distinct HPIV serotypes all of which are enveloped, negative–sense, ssRNA viruses belonging to the *Paramyxoviridae* family. The function and structure of the viral genes has been explored using mutant HPIV-1 viruses generated with a reverse genetics system [6], [7] or cell culture [8]–[10]. Evolutionary studies of HPIV-1 were performed in the 1990s using the hemagglutinin-neuraminidase (HN) and fusion (F) genes [11]–[14]. However, these studies are now dated and no evolutionary studies based on the complete genome of HPIV-1 have ever been conducted. Not only is there very little evolutionary data on HPIV-1whole genomes, but the publicly available sequence data for HPIV-1 is also extremely limited. To date there is only one complete genome sequence and 329 partial sequences. Many of these sequences are from mutant viruses created for the development of vaccines and therapeutic agents and therefore not useful for evolutionary analyses. Of the wild-type HPIV-1 sequences, most are partial sequences of the HN gene. Limited genomic data and poorly understood/studied HPIV-1 evolution has also been an impediment to the development of molecular diagnostics for HPIV-1. To address these gaps in knowledge and data, we developed a whole genome sequencing protocol for HPIV-1 from clinical samples and low passage virus isolates and report the genome sequences for 40 HPIV-1 strains. Thirty-nine of these strains were collected in Milwaukee, Wisconsin during 1997–1999, 2005–2007 and 2009–2010 and one was obtained from the American Type Culture Collection. Using these new data, we characterized the evolutionary pattern of HPIV-1 over the past 14 years in Milwaukee, WI and combined our data with previously published HN gene sequences from viruses collected from the USA and Japan over a 44-year period (1966–2010) to further examine the evolutionary pattern of HPIV-1.

## Materials and Methods

### Ethics Statement

All clinical samples were collected under protocols allowing sequencing approved by the Medical College of Wisconsin and Children's Hospital of Wisconsin institutional review boards. Some samples were approved to be collected retrospectively, de-identified, and did not require consent. The remaining samples were collected with written informed consent (from parent/guardian).

### Clinical Specimen and Isolates

Nasal swab specimens were collected from Children's Hospital of Wisconsin and its affiliated clinics. After collection, nasal swabs from each patient were immediately put into 3 mL of M4 viral transport medium (Remel, Lenexa, KS) and kept at 2–8°C. On the day of collection, 500 µL aliquots of each specimen were prepared and one aliquot from each specimen was tested in a one-step real-time RT-PCR assay for HPIV-1, -2, -3, -4 and human metapneumovirus (HMPV) that was developed in the clinical laboratory of the Midwest Respiratory Virus Program (MRVP). The remaining aliquots of each clinical specimen were frozen at −80°C for later use. Twenty-six HPIV-1 positive clinical specimens were collected during the 2009–2010 HPIV-1 season and used for this sequencing project. An additional 10 specimens collected during 1997–1999, one specimen collected in 2005, and two specimens collected in 2007 (using the same standard collection method), were retrieved from the MRVP virus bank. These 13 specimens tested positive for HPIV-1, at the time of collection, using the Hexaplex, a multiplex RT-PCR enzyme hybridization assay [15]. The C-35 strain, isolated in Washington D.C. in 1957, was obtained from American Type Culture Collection (ATCC, Rockville, MD). This strain was passaged 20 times at ATCC and one time in our lab. The passage history before the strain was sent to ATCC is not clear.

Because clinical specimens are a limited resource, all specimens, except for the 2005 and 2007 samples were inoculated onto Rhesus monkey kidney cells (LLC-MK2, ATCC, Rockville, MD) to propagate the viruses for future experiments. In brief, LLC-MK2 cells were cultured in 6-well plates at 37°C and 5% CO_2_ with Eagle's minimal essential medium (EMEM, Lonza, Walkersville, MD) containing 10% fetal bovine serum (HyClone, Logan, UT) and 1% Penicillin/Streptomycin (pen/strep) (Gibco, Grand Island, NY) until 90% confluence was achieved. Cells were washed twice with EMEM and then 100 µL of clinical specimen was combined with 100 µL of EMEM and added to each well. The plates were incubated at 37°C for 1 hour and gently rocked every 15 minutes to avoid drying of cells. Following incubation, 3 mLs of virus growth media [EMEM containing: 0.09% bovine serum albumin (BSA) (MP Biomedicals, Solon, OH), 2.4 µg/mL trypsin (Worthington, Lakewood, NJ), 1% pen/strep (Gibco) and 1% fungizone (Gibco)], was added into each well and the cells were returned to the incubator. Fifty µL of culture supernatant was used for a hemagglutination assay with guinea pig red blood cells (Colorado Serum Company, Denver, CO) on days 2, 4, 6, 8 and 10 post-inoculation. HPIV-1 viruses were harvested when the hemagglutination assay was positive. The supernatant from hemagglutination negative cultures was harvested at day 10 and blind passaged to new 6-well plates LLC-MK2 cells.

Of the 39 HPIV-1 viruses collected in Milwaukee, 10 were sequenced directly from clinical nasal swab specimens, 16 from low-passage (1–2 passages) clinical isolates and 14 were sequenced initially using clinical specimens, but gaps were filled in using low-passage isolates obtained from that specimen. Table 1 shows the pertinent information for each HPIV-1 strain including: sample type, the amount of sequence data available, and GenBank Accession number.

**Table 1:** Strain Information for all 40 HPIV-1 Genomes Sequenced.

GenBank Accession	Strain name	Collection date			Start to end of genomes	Sample type#
		Year	Month	Day		
JQ901971	HPIV-1/c35/1957 (ATCC VR-94)	1957	n/a	n/a	29-15550	I
JQ901975	HPIV-1/WI/629-001/1997	1997	10	3	29-15567	I and S
JQ901972	HPIV-1/WI/629-002/1997	1997	9	22	29-15539	I
JQ901976	HPIV-1/WI/629-003/1997	1997	10	16	76-15567	I
JQ901973	HPIV-1/WI/629-004/1997	1997	9	24	77-15560	I
JQ901974	HPIV-1/WI/629-005/1997*	1997	9	25	78-15555^a^	I
JQ901977	HPIV-1/WI/629-006/1997	1997	11	4	77-15560	I
JQ901979	HPIV-1/WI/629-007/1997	1997	11	22	28-15558	I
JQ901978	HPIV-1/WI/629-008/1997	1997	11	22	28-15568	I
JQ901980	HPIV-1/WI/629-009/1997	1997	12	3	29-15565	I
JQ901981	HPIV-1/WI/629-010/1999	1999	3	5	29-15549	I
JQ901982	HPIV-1/WI/629-A31/2005	2005	11	9	28-15555	S
JQ901983	HPIV-1/WI/629-003/2007	2007	n/a	n/a	29-15552	I
JQ901984	HPIV-1/WI/629-030/2007	2007	n/a	n/a	28-15534	I
JQ901985	HPIV-1/WI/629-001/2009	2009	n/a	n/a	27-15548	I
JQ901991	HPIV-1/WI/629-D01150/2009	2009	10	6	29-15568	S
JQ901987	HPIV-1/WI/629-D00057/2009*	2009	9	29	78-15485^b^	S
JQ901998	HPIV-1/WI/629-D00387/2009	2009	10	22	78-15557	I and S
JQ901989	HPIV-1/WI/629-D00712/2009	2009	10	2	29-15567	S
JQ902003	HPIV-1/WI/629-D01145/2009	2009	11	23	100-15548	I and S
JQ901996	HPIV-1/WI/629-D01164/2009	2009	10	20	100-15446	I and S
JQ901992	HPIV-1/WI/629-D01202/2009	2009	10	6	29-15568	S
JQ902006	HPIV-1/WI/629-D01250/2009*	2009	12	4	100-15539^C^	I and S
JQ902000	HPIV-1/WI/629-D01463/2009	2009	11	7	100-15481	I and S
JQ902004	HPIV-1/WI/629-D01575/2009	2009	11	30	100-15549	I and S
JQ901988	HPIV-1/WI/629-D01662/2009	2009	9	29	29-15539	S
JQ901986	HPIV-1/WI/629-D01681/2009	2009	9	29	27-15557	I
JQ902008	HPIV-1/WI/629-D01774/2009*	2009	10	3	81-15539^d^	I
JQ902001	HPIV-1/WI/629-D01790/2009	2009	11	7	100-15482	I and S
JQ901995	HPIV-1/WI/629-D01809/2009	2009	10	11	28-15539	I
JQ902007	HPIV-1/WI/629-D02039/2009	2009	12	10	100-15548	I and S
JQ902002	HPIV-1/WI/629-D02072/2009	2009	11	18	100-15544	I and S
JQ901993	HPIV-1/WI/629-D02130/2009	2009	10	6	29-15568	S
JQ901999	HPIV-1/WI/629-D02143/2009	2009	10	24	100-15492	I and S
JQ901990	HPIV-1/WI/629-D02161/2009	2009	10	3	29-15550	S
JQ902005	HPIV-1/WI/629-D02209/2009	2009	11	30	100-15488	I and S
JQ901994	HPIV-1/WI/629-D02401/2009	2009	10	6	29-15568	S
JQ901997	HPIV-1/WI/629-D01900/2009	2009	10	21	100-15492	I and S
JQ902010	HPIV-1/WI/629-D02071/2010	2010	3	2	100-15488	S
JQ902009	HPIV-1/WI/629-D02211/2010*	2010	2	15	100-15156^e^	I and S

* These sequences still have some gaps. Gene sequences with gaps in the coding region were not used for the coding region (ORF) analysis for that specific gene.

# The sample type indicates whether the genome was sequenced from a clinical isolate (I), a clinical specimen (S) or a combination of the two (I and S).

a Sequence of HPIV-1/WI/629-005/1997 has gaps at nts 8270–9308, 9520–10136, 10172–10175, 10640–10701, 11474–11883, 12475–12507 and 13196–13590.

b Sequence of HPIV-1/WI/629-D00057/2009 has gaps at nts 2199–2262, 8172–8653, 9750–10223, and 11823–11926.

c Sequence of HPIV-1/WI/629-D01250/2009 has gaps at nts 8271–8802.

d Sequence of HPIV-1/WI/629-D01774/2009 has gaps at nts 2301–2326, 3671–3772, 4070–4253, and 11906–13627.

e Sequence of HPIV-1/WI/629-D02211/2010 has gaps at nts 2995–3041, 3676–3740, 6408–7517, and 15157–15445.

### Primers for Amplification and Sequencing

The HPIV-1 genome was amplified by RT-PCR as ten overlapping fragments. Amplification primers were designed using an on-line primer design tool [IDT PrimerQuest (www.IDTDNA.com, Integrated DNA Technologies, Inc., Coralville, IA)] based on sequences retrieved from GenBank (primarily based on Accession # AF457102). Amplification primers were designed to, have melting temperatures of 55–60°C, have GC contents of around 50%, and amplify about 2 Kb of the HPIV-1 genome. The amplification primers were designed to cover only nucleotides (nt) 1–15575 of the 15600 nt genome as the low GC content at the extreme 5′ terminus makes primer design and amplification difficult.

The first and second fragments amplify similar regions of the genome and use the same reverse primer. The first fragment uses a forward primer that binds to the first nt of the genome; however this primer has a low GC content (37%) and only worked for 50% of the strains. The forward primer of the second fragment binds to the genome starting at nt 77. Strains that failed to produce an RT-PCR product for the first fragment were sequenced beginning slightly downstream of the 3′ terminus at the start of the second fragment.

The RT-PCR amplified fragments were sequenced with 44 primers. The amplification primers were also used as sequencing primers. All primers were synthesized by Integrated DNA Technologies (IDT) and are listed in Table 2. Those primers shown in bold print were used for both amplification and sequencing while those shown in plain print were used only for sequencing.

**Table 2:** HPIV-1 Primer Information for Amplification and Sequencing.

Name	Sequence*	% GC	Tm	Fragment
**P1F1−27**	**ACCAAACAAGAGGAAAAACTTGTTTGG**	37	56.8	1
**P1F77−23**	**AGGGACAAGTCACAGACATTTGA**	43.5	55.9	2
**P1R509+24**	**CATAGGTCCAAACAACCACTCTGT**	45.8	56.6	1 & 2
**P1F234−24**	**ACATTAGGCCCGAGTGTGACAGAT**	50	60	3
**P1R2336+24**	**TCCCTCAACTTGGTCTTCTTCCCT**	50	59.3	3
**P1F2208−24**	**ACCCTCTACCTGAGAATATGGGCA**	50	58.8	4
**P1R4352+24**	**CCTAGGTGTACCATGAAGTTGAGC**	50	56.9	4
**P1F4133−24**	**ACCTCAATGTCTCCCAGTCGACAA**	50	59.6	5
**P1R6538+24**	**TGTGCCATCCTCCAACTGCTGATA**	50	60	5
**P1F6277−24**	**GCGGGACAAACAGAATACCAGTGA**	50	58.9	6
**P1R8308+24**	**CACCTGATATGCATTCTCTCGGAC**	50	56.9	6
**P1F8123−23**	**CTTAGGTGCCGAAGGGAGGCTAC**	56.5	58.9	7
**P1R10202+24**	**TCCCATGCTGCTTTCCTAGGTGAT**	50	59.9	7
**P1F10061−24**	**GAATGCACAAGGATCCAACTCTGC**	50	58.2	8
**P1R11995+24**	**TCGTTGTATCAAGCATCCCGGCTA**	50	59.9	8
**P1F11751−24**	**TGGGCATCAGACCCTTATTCATGC**	50	59	9
**P1R13842+24**	**AGGATCCCAGTCCTCTAAGACTTC**	50	56.9	9
**P1F13501−24**	**TCTTTATGCGTGATTGGCAAGAGG**	45.8	57.6	10
**P1R15575+25**	**CCAGACAAGAGTTTAAGAAATATCG**	36	51.5	10
P1R278+24	GAGTGGGCTAAGAAAGTGGTTGCT	50	59	
P1F710−24	AGCATTCAGACAGGATGGAACCGT	50	60.1	
P1R1865+24	TTCGGCTTCAGGATCCCTCTCAAA	50	59.7	
P1F2783−24	ACCGCAAATGAAGAGGAAACCAGC	50	59.8	
P1R3764+27	CCTACCTTGACTATTCTGATGTGAGGG	48.1	57.8	
P1F4630−24	CTGTGTTCCAACCTGCAATTCCGA	50	59.6	
P1R5775+24	GAGAGATGATAATGCCTGCAAGGT	45.8	56.6	
P1F4455−24	TCTCTCGGGCTTGTAGGTGGAATA	50	58.8	
P1R6065+24	ACAGTTTGTGACATCTGCACCTCC	50	59.1	
P1F6803−24	TCCACAACTTCACCAATCAGGTGT	45.8	58.6	
P1F7677−23	TCTGTAATAGCTGCAGGAACAA	40.9	53.9	
P1R7856+26	CCACTTCCTACACTTGGATACATTGC	50	58.7	
P1F8740−21	ACTTAGGGTTAATGCCTGCCT	47.6	55.9	
P1F8513−24	TCATTTCGGGAAGGGCTACTGCTT	50	60.2	
P1R8819+23	TCGGAGAGTTCAAGTGACATTCT	43.5	55.7	
P1F9519−23	TCTTCAGCTGGACGACTCGATAA	47.8	57.2	
P1R9760+23	TGCTGTCTGCTTCTGAATCTGTG	47.8	57	
P1R9667+22	AGTTACAGGGTCATCCAACTGT	45.5	55.5	
P1F10576−24	CAGGTGTTCCAAGATCCAACTCAG	50	57.3	
P1R10701+23	TCGGTTGATGAGTCTGCAGCTTT	47.8	58.6	
P1R11565+24	ACCCTCCTATGTTAGCTGGGATCA	50	58.8	
P1F12388−24	GAAGTCCTGCCATACGTATCCCTT	50	58	
P1R13287+24	CCCATATTTCATCCCTCCCTCTGA	50	57.6	
P1F14090−25	CGGGATTAATAGCACCAGTTGTTTG	44	56.1	
P1R15017+23	ATCCGAGTCCACTCAAAGATTGT	43.5	55.8	

* Bold font represents oligonucleotides used for amplification. All primers were used for sequencing. The primer names are designed as follows: 1) The “P1” at the beginning indicates that these are HPIV-1 primers, 2) The “F” or “R” located at the third position of the primer name indicates whether the primer is in the forward or reverse orientation, 3) the number between the “F” or “R” and the hyphen corresponds to the nucleotide position of the 5′ end of the oligonucleotide in the HPIV-1 genome, and 4) adding or subtracting the number at the end of the primer name from the nucleotide position of the 5′ end of the oligonucleotide is the nucleotide position of the 3′ end of the oligonucleotide.

### Nucleotide Extraction, Amplification, Purification and Electrophoresis

Viral RNA was extracted from 400 µL of clinical specimen or tissue culture supernatant using the NucliSENS easyMAG (bioMérieux, Durham, NC) according to the manufacturer's protocol and eluted in 50 µL of elution buffer.

cDNA was synthesized in a 20 µL reaction mixture containing 2.5 mM random hexamers (Applied Biosystems, Carlsbad, CA), 4 mM dNTPs (Applied Biosystems), 4 mM MgCl_2_ (Applied Biosystems,), 1 U/µL RNase inhibitor (Applied Biosystems), 2.5 U/µL MuLv reverse transcriptase (Applied Biosystems) and 3 µL RNA. The reaction mixture was incubated for 5 minutes at 25°C and then for 14 minutes at 42°C followed by 1 minute at 95°C. Ten µL of cDNA was amplified by adding 40 µL of PCR mix containing a pair of PCR primers, 2.25 mM MgCl_2_, 2.5 U of Faststart hot start polymerase (Applied Biosystems) and the appropriate buffers to support PCR. The final concentration of each primer was 200 nM. Amplification was performed with a touch-down PCR program. After an initial denaturing at 94°C for 5 minutes, the PCR was done for 5 touch-down cycles with denaturing at 95°C for 45 seconds, annealing at 66°C for 5 minutes, and extension at 72°C for 90 seconds. The annealing temperature was decreased by 2°C after each cycle. This was followed by another 2 touch-down cycles with denaturing at 95°C for 45 seconds, annealing at 56°C for 2 minutes, and extension at 72°C for 90 seconds. Again, the annealing temperature was decreased by 2°C after each cycle. Finally, 30 standard PCR cycles were performed with denaturing at 95°C for 45 seconds, annealing at 52°C for 2 minutes and extension at 72°C for 90 seconds. The PCR was finished with a final extension at 72°C for 10 minutes.

The PCR products were purified with a Millipore MultiScreen PCR_μ96_ filter plate (Millipore, Billerica, MA) on the Biomek FX workstation (Beckman Coulter, Brea, CA) according to the Millipore PCR purification protocol. The purified PCR products were electrophoresed on an Agilent 2100 Bioanalyzer with a DNA 7500 kit and protocol (Agilent technologies, Inc., Santa Clara, CA) to determine the specificity and concentration of the amplified product.

### BigDye Sequencing Reaction and Analysis

The purified PCR products were diluted to ∼1 ng/µL based on the concentrations determined by the Agilent Bioanalyzer and used as the templates for sequencing reactions. The PCR products were sequenced with a BigDye terminator V.3.1 cycle sequencing kit (Applied Biosystems). Three µL of diluted PCR product was sequenced in a 10 µL reaction containing 0.5 µL BigDye terminator V.3.1 ready reaction mix, 1.35 µL of 5× sequencing buffer, 2 µL of 1.6 µM sequencing primer and 3.15 µL H_2_O. The sequencing reactions were cleaned up with a Millipore Montage SEQ_384_ sequencing reaction cleanup kit (Millipore) on the Biomek FX workstation following Millipore's protocol (Beckman Coulter). Finally the sequencing reactions were analyzed with the ABI 3730*xl* DNA analyzer (Applied Biosystems). Subsequent sequence data was assembled and proofread using the DNASTAR Lasergene 8 software (DNASTAR, Madison, WI).

### Phylogenetic Analysis

The HPIV-1 genomes obtained from this study were analyzed using both the whole genome and also the individual genes. Among the 40 genome sequences obtained in the current study, five sequences (from isolates HPIV-1/WI/629-005/1997, HPIV-1/WI/629-D00057/2009, HPIV-1/WI/629-D02211/2009, HPIV-1/WI/629-D01774/2009, and HPIV-1/WI/629-D01250/2009) had gaps in coverage of some of the open reading frames (four had gaps in the HN gene). These sequences were excluded from phylogenetic analyses in which they did not have the complete genetic sequence (except in the case of the whole genome analysis). In addition, publicly available sequences from GenBank were also added to each analysis. Only those GenBank sequences obtained from wild-type HPIV-1 viruses that were greater than 100 bp in length were used for phylogenetic analyses.

Sequence alignments for the ORFs of individual genes were constructed using the Se-Al program (Rambaut et al., 1996). Because the collection year is known for most of the HPIV-1 sequences (for both the current strains and those from GenBank) and regression analysis using the program Path-O-Gen (Version 1.3) showed that there is clock-like evolution (data not shown), we used the Bayesian Markov Chain Monte Carlo (MCMC) method with a strict molecular clock in the BEAST program (Version 1.6.1) [16] to infer the time-scaled evolutionary relationships for HPIV-1. One of the 222 HN gene sequences in GenBank was missing collection date information so only 221 additional HN gene sequences were used for this analysis. The analysis incorporated the GTR model of nt substitution, with a different substitution rate estimated for each codon position. The chain length was set to 5 million. Trees were sampled every 1000 generations. The Maximum Clade Credibility (MCC) tree generated by BEAST was analyzed using TreeAnnotator (v 1.6.1, available at http://beast.bio.ed.ac.uk) with 10% burn-in. Statistical uncertainly is reflected in values of the 95% Highest Probability Density (HPD). The trees were viewed in FigTree (1.3.1, available at http://beast.bio.ed.ac.uk). Each clade is defined by long branches and nodes supported by high Bayesian posterior probability (BPP) values (>0.9). Similar results were obtained using a relaxed clock model (SRD06 model, Bayesian Skyline prior, run 100 million generations) (Data not shown).

In addition to the analyses done with the BEAST program, the phylogenies for each gene were also inferred using the maximum likelihood method in PAUP [17] with high bootstrap (>70%). For all of the genes other than the HN gene, only those sequences covering the entire coding region of the gene were used. For the HN gene, the phylogeny was inferred using nt positions 7245–8479 (nts 343–1577 of the 1728 nt HN gene) because this was the sequence range in which those HN gene sequences from our study and GenBank (used in the MCMC analysis) were primarily complete. The best-fit model of nucleotide substitution was tested and selected by MODELTEST [18] as the general reversible model (GTR+I+G) with the frequency of each substitution, proportion of invariant sites (I) and the gamma distribution of among-site rate variation with four rate categories. The bootstrap resampling process (1,000 replications) was performed using the neighbor-joining (NJ) method to assess the robustness of each node on the tree.

### Amino Acid Substitution among Clades

The parsimony-based MacClade program [19] was used to determine amino acid changes in the proteins translated from the N, P, M, F, HN, and L genes.

### Analysis of Nucleotide Diversity

Analyses of nucleotide diversity were performed using DNAsp version 5.10.01 [20]. The initial analysis was done using all of the genetic data obtained from this study plus the one HPIV-1 complete genome sequence that is currently available in GenBank (AF457102). This analysis showed that the HPIV-1 genome has a nucleotide diversity (*π* = the average number of nucleotide differences per site between two sequences) of 0.019 across the entire genome. However, 6,150 nts were excluded from this analysis because DNAsp does not include nt positions in which any sequence has missing data when performing nucleotide diversity analyses. After removing HPIV-1/629-005/1997, HPIV-1/629-D00057/2009, HPIV-1/629-D01250/2009, HPIV-1/629-D01774/2009 and HPIV-1/629-D02211/2010, which still have some sequence gaps, from the analysis, the nucleotide diversity was 0.018 and only excluded 273 positions. The similar nucleotide diversities indicates that the remaining 35 sequences are likely an accurate representation of what would be seen if all 40 sequences had complete genetic data. With this in mind we believe that using fewer, but more complete, sequences actually allowed us to perform a more accurate analysis of the genetic diversity of the HPIV-1 genome and performed all nucleotide diversity analyses with only the 35 complete genome sequences. Beside analysis of the complete genomes, nucleotide diversity was also determined for each non-coding region, gene, and intergenic region.

Genetic diversity was also calculated using a sliding scale analysis across the whole genome. The sliding scale analysis calculates the nucleotide diversity across a 100 bp window rather than across an entire section of the genome (e.g. an entire gene). The window was moved in 25 nt increments across the entire HPIV-1 genome and the nucleotide diversity was recalculated for each window. The population's mutation rate was estimated as Theta/site (from S) (the Watterson Estimator). Finally, the ratio of non-synonymous (Ka) to synonymous (Ks) mutations was also calculated in DNAsp using only the coding portions of the genome.

### Recombination Analysis

The 40 HPIV-1 genomes reported here and the genome sequence AF457102 retrieved from GenBank were analyzed for possible recombination using Recombination Detection Program v.3.31 [21]–[27] with three analysis methods: RDP, GENECONV, and BootScan.

## Results

### HPIV-1 Genome Sequencing Protocol

We have developed an RT-PCR protocol capable of amplifying nucleotides (nts) 1–15575 of the 15600 nt genome of HPIV-1 in ten overlapping fragments. Following amplification it is possible to obtain an accurate sequence of the entire HPIV-1 genome minus approximately 30–100 nts at either end. Forty HPIV-1 strains including clinical specimens and low passage virus isolates were sequenced using the sequencing protocol developed in this study and complete genomes were obtained for 35 of 40 HPIV-1 strains (Table 1). All 40 HPIV-1 sequences have been submitted to GenBank (accession numbers: JQ901971–JQ902010). The genome of HPIV-1 consists of the N-P(C/Y)-M-F-HN-L genes separated by intergenic sequences and flanked by a 5′ and 3′ non-coding region. The HPIV-1 intergenic sequence is CTT between the N-P, P-M, F-HN, and HN-L genes and is CGT between M-F genes. These three intergenic nts were highly conserved with no changes observed in sequences from either GenBank or this study. The terminators at the end of the HPIV-1 mRNAs are also highly conserved and similar among different genes. The sequence motif of the terminators is WADTAAGAAAAA. The third nt varies among A/T/G while the first position is consistently an A except in the L gene terminator where it is changed to a T. Also, a six nt addition was found from nt 4896–4901 of HPIV-1/WI/629-D01575/2009.

### Phylogenetic analysis of HPIV-1 in Milwaukee, WI since 1997

**Figure 1:**
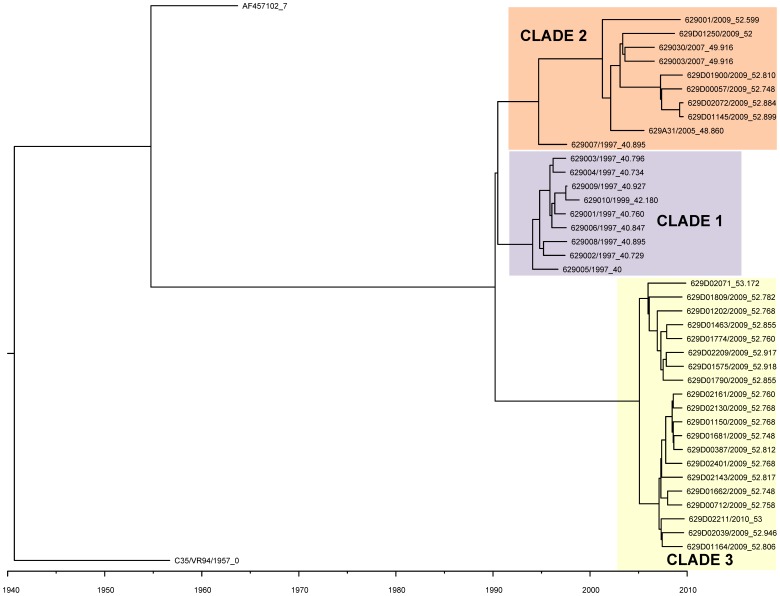
The phylogenetic relationships of the 40 genomes and sequence AF457102 using the BEAST program. The phylogeny of 40 recently sequenced HPIV-1 genomes and one sequence from GenBank (AF457102) was estimated using a Bayesian Markov Chain Monte Carlo (MCMC) method with a strict molecular clock. Strain HPIV-1/c-35/1957 showed the greatest distance from the 39 1997–2010 Milwaukee viruses which is consistent with its isolation time. Colored rectangles (labeled clade 1–3) represent the three clades of the 39 1997–2010 Milwaukee viruses. The sequence from strain HPIV-1/WI/629-007/1997 is a singleton. The scale bar shows the unit for branch age. The numbers following the underscore in each name represent the collection date in number of years since collection date of the oldest HPIV-1 strain. To make the figure more legible identical sequences were removed from the table.

The Bayesian Markov Chain Monte Carlo (MCMC) analysis of 39 HPIV-1 genome sequences from viruses collected in Milwaukee, WI during 1997–2010 identified three major HPIV-1 clades, referred to as Clades 1, 2, and 3 (Figure 1). Clade 1 includes viruses from 1997–1999, Clade 2 includes viruses from 2005–2009, and Clade 3 includes the majority of 2009 viruses and all of the 2010 viruses. It is interesting to note that the HPIV-1 viruses in clade 2 are more closely related genetically to viruses from clade 1 (collected in 1997–1999), despite being closer temporally to the viruses from clade 3.The Bayesian MCMC analysis indicates that the Time to the Most Recent Common Ancestor (TMRCA) is 66.7–72.0 years (95% HPD). The rate of evolutionary change for these viral genomes is 7.61×10^−4^ nt substitutions per site per year (95% HPD, 6.93×10^−4^ to 8.29×10^−4^ nt/site-year), which is within the range typically observed for negative-sense RNA viruses [28].

**Figure 2:**
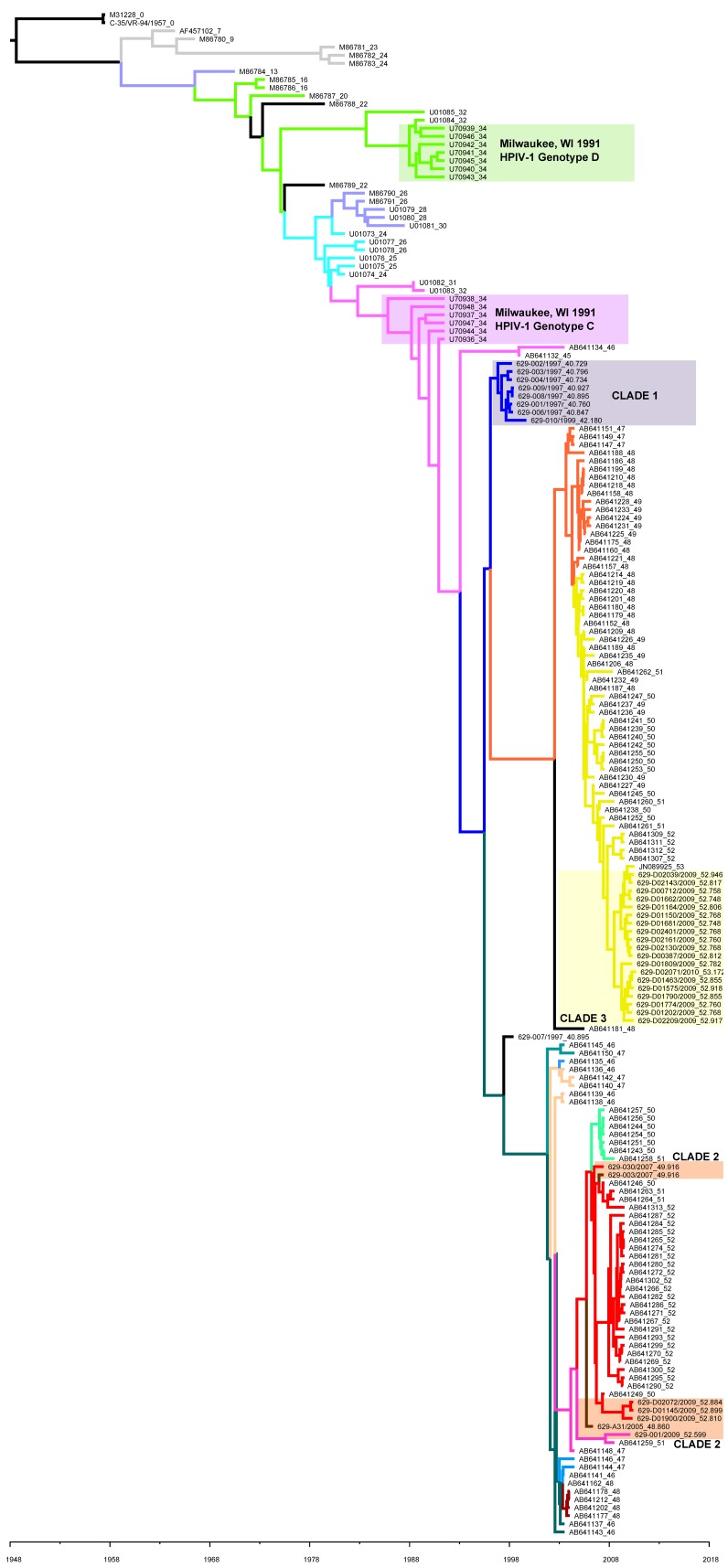
The phylogenetic relationships of HN genes including 37 present sequences and 221 sequences from GenBank. The phylogeny of 258 HN gene sequences was analyzed using a Bayesian Markov Chain Monte Carlo (MCMC) method with a strict molecular clock in the BEAST program. Colored rectangles labeled clades 1–3 represent the three clades identified in Figure 1 containing the 36 HPIV-1 sequences from the present study. Colored rectangles labeled genotypes C and D represent the HN gene sequences from HPIV-1 viruses collected in Milwaukee, WI in 1991. The number following the underscore in each name represents the collection date in years since collection date of the oldest HPIV-1 strain. Branch color corresponds to clade name/HN sequence seen in Figure 3.

A second MCMC tree was made using 36 of the 40 newly identified HN gene sequences (four were excluded because they were incomplete) plus 221 HN gene sequences deposited in GenBank from previous studies [7], [12]–[14], [29] (Figure 2). The phylogeny was structured both by time and by location of isolation. Clades 1 and 3 identified during the whole genome phylogeny were also evident on the HN phylogeny. However, the viruses belonging to clade 2 (as defined by whole genome phylogeny in Figure 1) were interspersed with viruses from Japan on the HN gene tree. The TMRCA for the HN gene is estimated to be 58.3–66.2 (95% HPD), which is similar to the estimate for the complete genomes from Milwaukee 1997–2010. The rate of evolution for the HN gene is 1.37×10^−3^ nt substitutions per site per year (95% HPD: 1.16×10^−3^ nt/site-year to 1.59×10^−3^ nt/site-year, similar to the rest of the genome.

In an effort to confirm the results of the BEAST analysis we also used the maximum likelihood method available in PAUP (version 4.0) [17]. The phylogenies created using the 40 newly identified HPIV-1 whole genomes and the N, P, M, F, HN and L gene sequences (Figures S1, S2, S3, S4, S5, S6, S7, S8) all look very similar to the MCMC tree created with complete HPIV-1 genomes in BEAST (Figure 1). The viruses are grouped in the same clade regardless of which gene is being analyzed. Figures S1, S2, S3, S4, S5, S6, S7, S8).

### Amino Acid Substitutions

**Figure 3:**
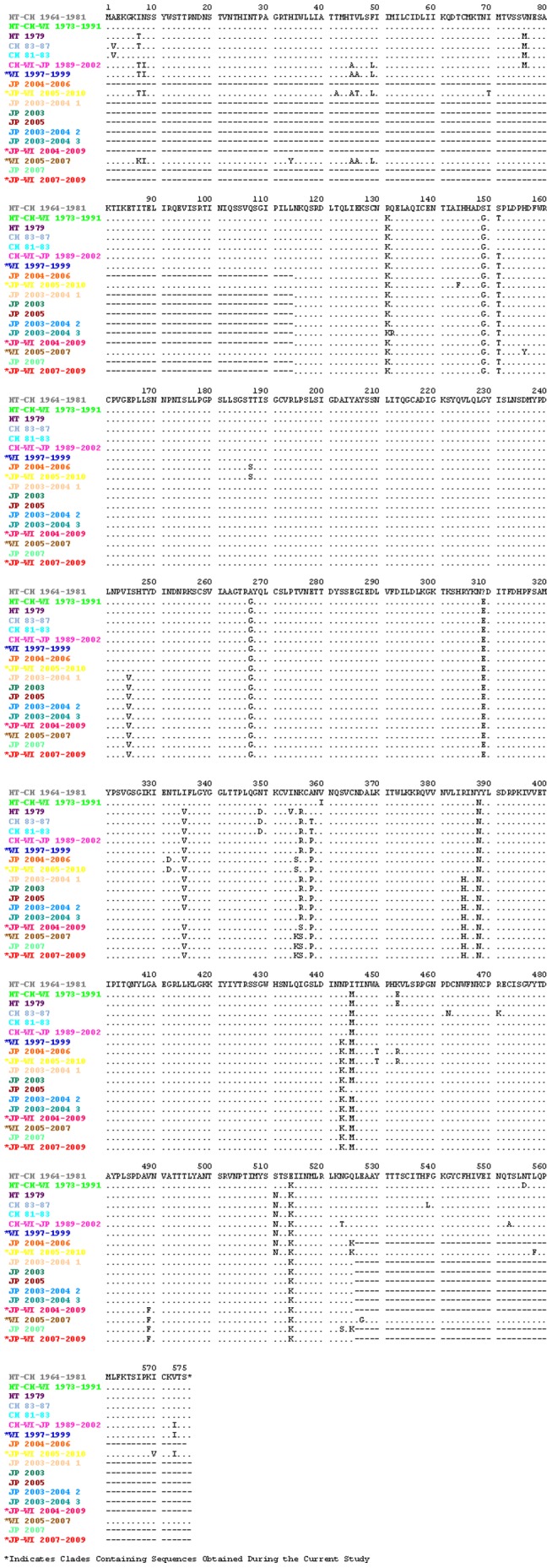
Amino acid sequence of the HN protein across all HPIV-1 clades. This figure shows the amino acid sequence of the HN protein across all HPIV-1 clades. Amino acid substitutions were determined with the MacClade program and the consensus HN gene sequence for each clade. The color of the branches in Figure 2 correspond to the color of the clade name in this figure indicating which viruses have each of the sequences listed above.

Amino acid substitutions were identified using the 40 newly sequenced genomes and the MacClade program (Maddison and Maddison, 2000) for each HPIV-1 protein by translating the open reading frame (ORF) of each gene. Except for a few singleton mutations, most amino acid substitutions are clade-scale changes (substitutions occur in all strains within a clade). We observed substitutions in 1 of 525 (0.2%) amino acids of the N protein, 25 of 569 (4.4%) amino acids of the P protein, 0 of 349 (0.0%) amino acids of the M protein, 6 of 556 (1.1%) amino acids of the F protein, 36 of 576 (6.3%) amino acids of the HN protein, and 13 of 2224 (0.6%) amino acids of the L protein. The clade-scale amino acid substitutions are listed in Table 3 (complete genome sequences from this study) and shown graphically in Figure 3 (HN gene sequences from this study and those found in GenBank).

**Table 3:** Clade-Scale Amino Acid Substitutions from the N, P, M, F and L Proteins of 40 Recently Sequenced HPIV-1 Complete Genomes (the HN protein is described in greater detail in Table 4 and is not included here).

Protein	Substitutions by clades			Multiple substitutions
from	Clade 1	Clade 2	Clade 3	
**N gene**	None	None	N498S	None
**P gene**	N56S, P123S, P125L,	G110D, D185N, P231Q, S247P, K270R, T282A, V534A	N70D, I90V, I111V, P141S, S162F, P182S, T267I, S268G, S/P274L, S321G, S324P, V534A	F8S/L^a^, L151P/S^a^, G473S/T^a^
**M gene**	None	None	None	None
**F gene**	E5K	N321S	T493K, V526I, R546K	F8L/I^a^
**L gene**	M718I, L1605F	N199D, I210F, V2165I	Q4L, V93I, T728A, I1176V, V1506I, N1598H, K1747R, T2209I	None

a These residues have more than two amino acid variations.

When using the 40 new HN gene sequences in addition to those available in GenBank we found that 48 of 576 amino acids in the HN gene were variable (8.3%) (Table 4). The HN protein is comprised of seven regions [30]. Across these seven regions, the cytoplasmic tail (residues 1–35), the transmembrane region (residues 35–60), the stalk region (residues 60–191), the high structural homology region I (residues 191–341), the non-structural assignment region (residues 341–457), the high structural homology region II (residues 457–504) and the carboxyl terminus (residues 504–575) showed 4, 4, 9, 5, 12, 3, and 11 amino acid substitutions, respectively. The transmembrane and carboxy terminal regions showed the highest substitution rates (0.160 and 0.155 substitutions/residue, respectively), while the high structural homology regions I and II were the most conserved [0.033 and 0.064 substitutions/residue, respectively (Figure 3, Table 4)].

**Table 4:** Amino Acid substitutions of the Seven Regions of HN Gene (calculated using all available sequences from GenBank).

Region Name	Location of region	Length of the region (Residues)	No. of Substitutions in each region	Ratio of substitution/residue
**Cytoplasmic tail**	1–35	35	4	0.114
**Transmembrane region**	36–60	25	4	0.160
**Stalk region**	61–191	131	9	0.069
**High structural homology region I**	192–341	150	5	0.033
**Non-structural assignment region**	342–457	116	12	0.103
**High structural homology region II**	458–504	47	3	0.064
**Carboxyl terminal**	505–575	71	11	0.155

### Analysis of Sequence Variability

The nucleotide diversity (*π*) is 0.018 across the entire genome (using the 35 complete genomes obtained in this study and the one complete genome previously available in GenBank). Nucleotide diversity was also calculated for important genetic elements of HPIV-1 including: 5′ and 3′ non-coding regions (NCRs), intergenic sequences, gene terminators, and open reading frames (ORFs). All of the non-coding regions were well conserved with the exception of the 5′ NCR of the F gene (π = 0.050) and 3′ NCR of the HN gene (π = 0.050), which were the most diverse regions of the genome. A 6 nt addition was found in the 5′ NCR of the F gene of HPIV-1/WI/629-D01575/2009. Among the six ORFs of the HPIV-1 genome, the P gene is the most variable (π = 0.023) and the L gene is the most conserved (π = 0.014) (Table 5).

**Table 5:** Genetic Diversity Across the HPIV-1 Genome.

Region	Positions	Sites analyzed	Polymorphic (Segregating) Sites	Average nucleotide differences (*k*)	Nucleotide Diversity (*π*)	Theta/Site from S
5′ Leader	1–55	N/A^a^	0	0.000	0.000	0.000
N 5′ NCR^b^	56–116	17^b^	7^b^	1.319^b^	0.078^b^	0.099
N Gene	120–1694	1575	157	23.444	0.015	0.024
N 3′ NCR	1695–1737	43	7	1.438	0.033	0.039
N/P – IG Spacer	1738–1740	3	0	0.000	0.000	0.000
P 5′ NCR	1741–1843	103	13	2.073	0.020	0.030
P Gene	1844–3550	1706	233	39.595	0.023	0.033
P 3′ NCR	3551–3633	83	14	2.454	0.030	0.041
P/M – IG Spacer	3634–3636	3	0	0.000	0.000	0.000
M 5′ NCR	3637–3668	32	5	0.383	0.012	0.038
M Gene	3669–4715	1046	113	17.873	0.017	0.026
M 3′ NCR	4716–4809	94	16	2.506	0.027	0.041
M/F – IG Spacer	4810–4812	3	0	0.000	0.000	0.000
F 5′ NCR	4813–5087	269	80	13.433	0.050	0.072
F Gene	5088–6755	1667	179	29.105	0.017	0.026
F 3′ NCR	6756–6843	88	17	2.433	0.028	0.047
F/HN – IG Spacer	6844–6846	3	0	0.000	0.000	0.000
HN 5′ NCR	6847–6902	56	14	1.663	0.030	0.060
HN Gene	6903–8630	1728	209	35.375	0.020	0.029
HN 3′ NCR	8631–8740	109	23	5.429	0.050	0.051
HN/L – IG Spacer	8741–8743	3	0	0.000	0.000	0.000
L 5′ NCR	8744–8771	28	1	0.056	0.002	0.009
L Gene	8772–15443	6668	588	96.384	0.014	0.021
L 3′ NCR	15444–15543	3^b^	1	0.356	0.119^b^	0.080
Tail Sequence	15544–15606	N/A^a^				
WHOLE GENOME	1–15606	15333	1677	275.319	0.018	0.026

a Sites could not be analyzed due to missing sequence data.

b Partial sequence of this region was not analyzed due to missing sequence data. The calculated nucleotide difference and diversity is not the accurate representation of the 5′ NCR.

**Figure 4:**
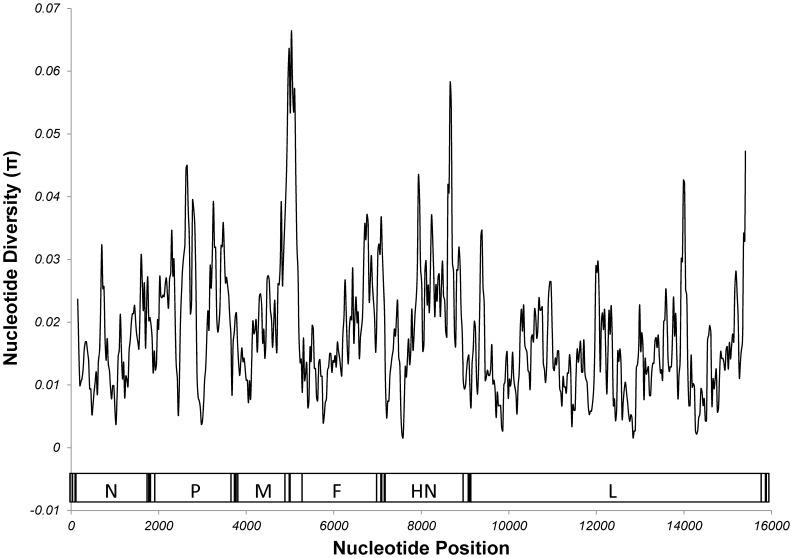
Sliding Scale Analysis of Nucleotide Diversity of the HPIV-1 Genome. Analysis of nucleotide diversity was done in 100 nt windows in 25 nt increments. A table showing each window and its diversity can be seen in supplementary document 1. A schematic of the HPIV-1 genome can be found at the bottom of the figure. The coding region of each gene is labeled.

The complete results of the sliding scale analysis of nucleotide diversity can be seen in Figure 4. The 100 bp regions containing the 5′ NCR plus the first 5 nt of the F gene (nt 4983–5082) and the 3′ NCR of the HN gene (nt 8609–8709) showed the greatest nucleotide diversity (π = 0.066, π = 0.058) across the entire genome. The most diverse region of each open reading frame is from nts 650–749 (π = 0.032) for the N gene, 2600–2699 (π = 0.045) for the P gene, 4452–4551 (π = 0.027) for the M gene, 5083–5182 (π = 0.042) for the F gene, 7884–7983 (π = 0.042) for the HN gene, and 13939–14038 (π = 0.043) for the L gene (Figure 4).

### Analyses of Selective Pressure

The Ka/Ks ratio for the coding region of each gene ranges from 0.034–0.233 (values less than one are indicative of purifying/negative selection). The P gene has the greatest Ka/Ks ratio of all HPIV-1 genes (0.233) and the M gene has the smallest Ka/Ks ration (0.034).

### Recombination Analysis

Finally, we used the Recombination Detection Program (RDP) to identify possible recombination events among the 40 whole-genome HPIV-1 sequences. This analysis indicated that none of the strains that we sequenced exhibited any signs of recombination, which is consistent with the phylogenetic analyses performed in this study (Figures S1, S2, S3, S4, S5, S6, S7, S8).

## Discussion

By developing a novel PCR-based complete genome sequencing protocol for HPIV-1, we were able to provide the first evolutionary analysis of HPIV-1 at the complete whole genome level. Thirty-nine HPIV-1 genome sequences were obtained directly from clinical nasal swab specimens and/or viral isolates from 1997–1999, 2005, 2007 and 2009–2010 collected in Milwaukee, WI (and an additional sequence was obtained from an HPIV-1 isolate from ATCC). These data were combined with HPIV-1 gene sequences publicly available in GenBank.

Previous evolutionary studies of HPIV-1, based on the HN gene sequence, indicate that evolution is guided by a combination of co-circulating strains and the development of geographically restricted lineages [12]–[14]. The sequences used for the present analysis include those used in previous analyses and a significant number of additional sequences, which increases the overall temporal and geographic distribution of HPIV-1 sequence data. Previous studies of HPIV-1 in Milwaukee, WI revealed that two distinct HPIV-1 genotypes were circulating in Milwaukee during 1991 (genotypes C and D), confirmed by this study (Figure 2 and Figure S7) [12]. The current study indicates that two distinct HPIV-1 viruses continue to circulate in Milwaukee, WI; however, both are derived from genotype C. Even though no HPIV-1 genotype D viruses were detected, it remains possible that HPIV-1 genotype D may still be present in different parts of the country or even in WI, but if it has stopped circulating it would be interesting to look at nt polymorphisms or amino acid substitutions specific to this genotype to see if they may have contributed to its extinction (e.g. HN amino acid substitutions T7I and M445I).

We inferred the phylogenies of the HPIV-1 complete genome and the HPIV-1 HN gene using Bayesian Markov Chain Monte Carlo analysis (MCMC) with current sequences and those available in GenBank. The analyses showed that the topology of the whole genome tree was similar to that seen for the HN gene and supported the same three clade topology for the 1997–2010 Milwaukee viruses. While the TMRCA estimated for the HN gene and the whole genome were similar [58.3–66.2 years (95% HPD) and 66.7–72.0 years (95% HPD), respectively], the substitution rates obtained with these two analyses differed slightly (1.37×10^−3^ nt substitutions per site per year and 7.61×10^−4^ nt substitutions per site per year, respectively). With everything being equal this result indicates that the HN gene evolves more rapidly than the rest of the genome. However, the HN gene analysis contained nearly six times the number of sequences from a greater temporal and geographic distribution, and the difference in substitution rates is more indicative of sequence availability rather than a true difference in evolutionary rate. With that being the case, these results suggest that the HN gene evolves in a manner similar to that of the whole genome and that the HN gene may be an appropriate model for HPIV-1 evolution when whole genome sequences are not available. In order to get a better idea whether this finding is appropriate we would need to look at additional HPIV-1 whole genomes from different geographic locations.

In addition to a broad overview of the evolutionary pattern seen in HPIV-1 we also performed more in depth analyses on the HPIV-1 genome sequences by looking at nucleotide diversity and selection pressure. As was mentioned previously, an analysis of nucleotide diversity revealed that the 5′ NCR of the F gene and the 3′ NCR of the HN gene were the most diverse non-coding regions of the genome. Previous reports indicate that the 5′ NCR of the F gene contains cis-acting elements that affect transcriptional termination [31] and may regulate the level of read through transcription across the M-F junction and subsequent F protein production [32]. We also looked at the diversity in the coding regions of the HPIV-1 genome using both analyses of nt substitution [nucleotide diversity (π)] and amino acid substitution [(non-synonymous/synonymous substitution rate (Ka/Ks)]. The non-synonymous/synonymous (Ka/Ks) mutation ratios of less than 1 that is seen in all HPIV-1 genes indicates that, as a whole, HPIV-1 proteins are under purifying/negative selection. However, some proteins seem much more tolerant of amino acid substitutions than others. For example, it is interesting to note that the Ka/Ks ratios for the HN and P genes are approximately 3–8 times greater than the ratios for all other HPIV-1 genes (Table 6), which may indicate that these genes are more tolerant of non-synonymous mutations.

**Table 6:** Nonsynonymous (Ka) vs. Synonymous Mutations in the Coding Region of HPIV-1 Genes.

Region	Positions	Average Ka	Average Ks	Ka/Ks
N Gene	120–1694	0.002	0.060	0.040
P Gene	1844–3550	0.014	0.061	0.233
M Gene	3669–4715	0.002	0.067	0.034
F Gene	5088–6755	0.004	0.062	0.071
HN Gene	6903–8630	0.011	0.057	0.185
L Gene	8772–15443	0.002	0.060	0.041
ALL GENES	1–15606	0.005	0.060	0.083

At first blush it seems counterintuitive that a protein, such as the HN, with so many important functions would be among the most tolerant of amino acid substitution. One previous study reports that residues 184, 262, 264, 277, 279, 420, 461, and 541 play a role in hemaglutinase and neuraminidase function, while another indicates that residue 55 plays a key role in envelope fusion, and yet another describes how the conserved cysteine and proline residues around residue 461 are important to the viral structure [9], [10], [33]. However, as a surface protein, and one of the primary immunogens of HPIV-1, an increased tolerance of non-synonymous mutations outside of these key functional domains would likely aid the virus' ability to escape the host immune response. In support of this hypothesis, the current study shows that the residues previously identified as having a functional role showed little to no variation over the last 30 years while the remainder of the protein is more tolerant of amino acid substitution.

The P gene region encodes the P, C, C', Y1 and Y2 proteins. These viral accessory proteins aid the L protein in transcription/translation of viral nucleic acid and also aid in suppression of the host innate immune response by acting as antagonists to the IFN and apoptosis pathways [34]. The rationale for an increased Ka/Ks ratio in the P protein might be linked to the function of these accessory proteins that would benefit from increased diversity.

While the F gene showed less overall diversity than either the HN or P gene (in terms of nucleotide diversity and Ka/Ks ratio), portions of the gene are among the most conserved and the most diverse in the whole genome. Nts 5383–6107 are among the least tolerant of mutation, which is somewhat expected as this region contains the previously identified F protein cleavage site (the QTRFFG motif seen at F protein residues 110–115 was completely conserved among all 40 HPIV-1 sequences from this study) [11]. Meanwhile, nts 6233–6382 are among the most tolerant of mutation. As with the HN protein, the F protein is an important virus surface protein and increased variation in certain regions of the F protein may help the virus avoid the immune system.

Despite the N gene/protein being among the most conserved in HPIV-1, we did find a hypervariable region at the C-terminus of the N protein. Studies of Sendai virus found that this region is required for proper template function during RNA synthesis [35]. The N protein of Sendai virus and HPIV-1 share common structures and functional sites and as such the variable regions found in the N protein of HPIV-1 might have a similar function in RNA synthesis. The substitutions found at residues 442 have been documented previously in a mutant virus [36]–[38]. In this study, we show that these substitutions are common in all three clades of 1997–2010 Milwaukee HPIV-1 viruses and that they have remained consistent for over a decade.

Finally, we performed an analysis to see if any of these viruses have undergone recombination. Natural recombination events have been detected in several other members of the *Paramyxoviridae* family including Newcastle disease virus (NDV) [39]–[42] and human respiratory syncytial virus [43]. Attenuated vaccines are capable of influencing the evolution process of NDV through exchanging their genetic material with circulating viruses [44]. Recently, Yang et al [27] identified a naturally occurring HPIV-3 recombinant virus. In our recombination analysis of the 40 genomes reported in this study, we found no indication that any recombination events occurred. This result seems logical considering the fact that the topology of phylogenetic trees for each HPIV-1 gene group together in a nearly identical manner.

This work represents a significant step forward in filling the void of much needed HPIV-1 genetic data. Our hope is that the genome sequencing protocol we provide here will facilitate an even greater increase in HPIV-1 genetic data from diverse locales and time periods. This increase in genetic data may in turn lead to breakthroughs in the development of novel HPIV-1 therapeutics, vaccines and diagnostic assays. In the meantime, the genetic data provided by this study should lead to an increased understanding of HPIV-1 evolution, gene structure and function, viral pathogenesis, and conserved domains that can function as targets for molecular diagnostic assays.

## Supporting Information

Figure S1 **Phylogenetic relationship of HPIV-1 whole genomes with the maximum likelihood method in PAUP.** The accession number of the sequence from GenBank is AF457102.
(TIF)

Figure S2 **Phylogenetic relationship of HPIV-1 N gene including five sequences from GenBank with the maximum likelihood method in PAUP.** The accession nos. of the five GenBank sequences are: D01070, EU346886, M62850, S38060 and AF457102.
(TIFF)

Figure S3 **Phylogenetic relationship of HPIV-1 P gene including five sequences from GenBank with the maximum likelihood method in PAUP.** The accession nos. of the five GenBank sequences are: M37792, M74080, M74081, M74082, and AF457102.
(TIFF)

Figure S4 **Phylogenetic relationship of HPIV-1 M gene including three sequences from GenBank with the maximum likelihood method in PAUP.** The accession nos. of the three GenBank sequences are: M80818, S38067 and AF457102.
(TIFF)

Figure S5 **Phylogenetic relationship of HPIV-1 F gene including three sequences from GenBank with the maximum likelihood method in PAUP.** The accession nos. of the three GenBank sequences are: AF016279, AF457102 and M22347.
(TIFF)

Figure S6 **Phylogenetic relationship of HPIV-1 HN gene using sequences from the current study only with the maximum likelihood method in PAUP.**
(TIFF)

Figure S7 **Phylogenetic relationship of HPIV-1 HN gene including 221 sequences from GenBank with the maximum likelihood method in PAUP.**
(TIFF)

Figure S8 **Phylogenetic relationship of HPIV-1 L gene including two sequences from GenBank with the maximum likelihood method in PAUP.** The accession nos. of the two GenBank sequences are: AF117818 and AF457102.
(TIFF)
