# Supplementary figures and images for: Genome Sequencing and Phylogenetic Analysis of 39 Human Parainfluenza Virus Type 1 Strains Isolated from 1997–2010
Source: PLoS One. 2012 Sep 27;7(9):e46048. doi: 10.1371/journal.pone.0046048 (PMC3459887; doi:10.1371/journal.pone.0046048)

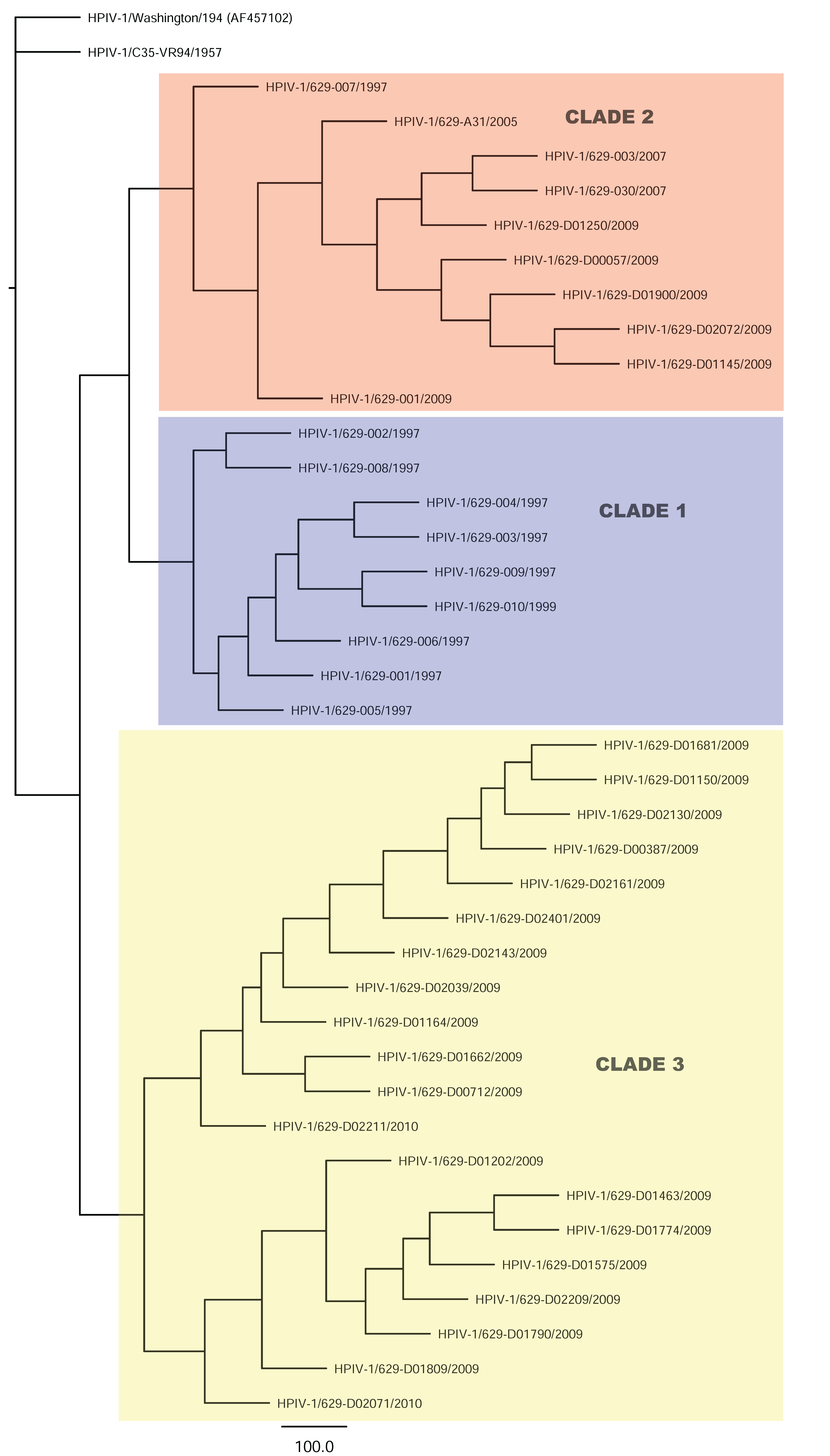

Supplement: Figure S1 — Phylogenetic relationship of HPIV-1 whole genomes with the maximum likelihood method in PAUP. The accession number of the sequence from GenBank is AF457102. (TIF) [file pone.0046048.s001.tif]

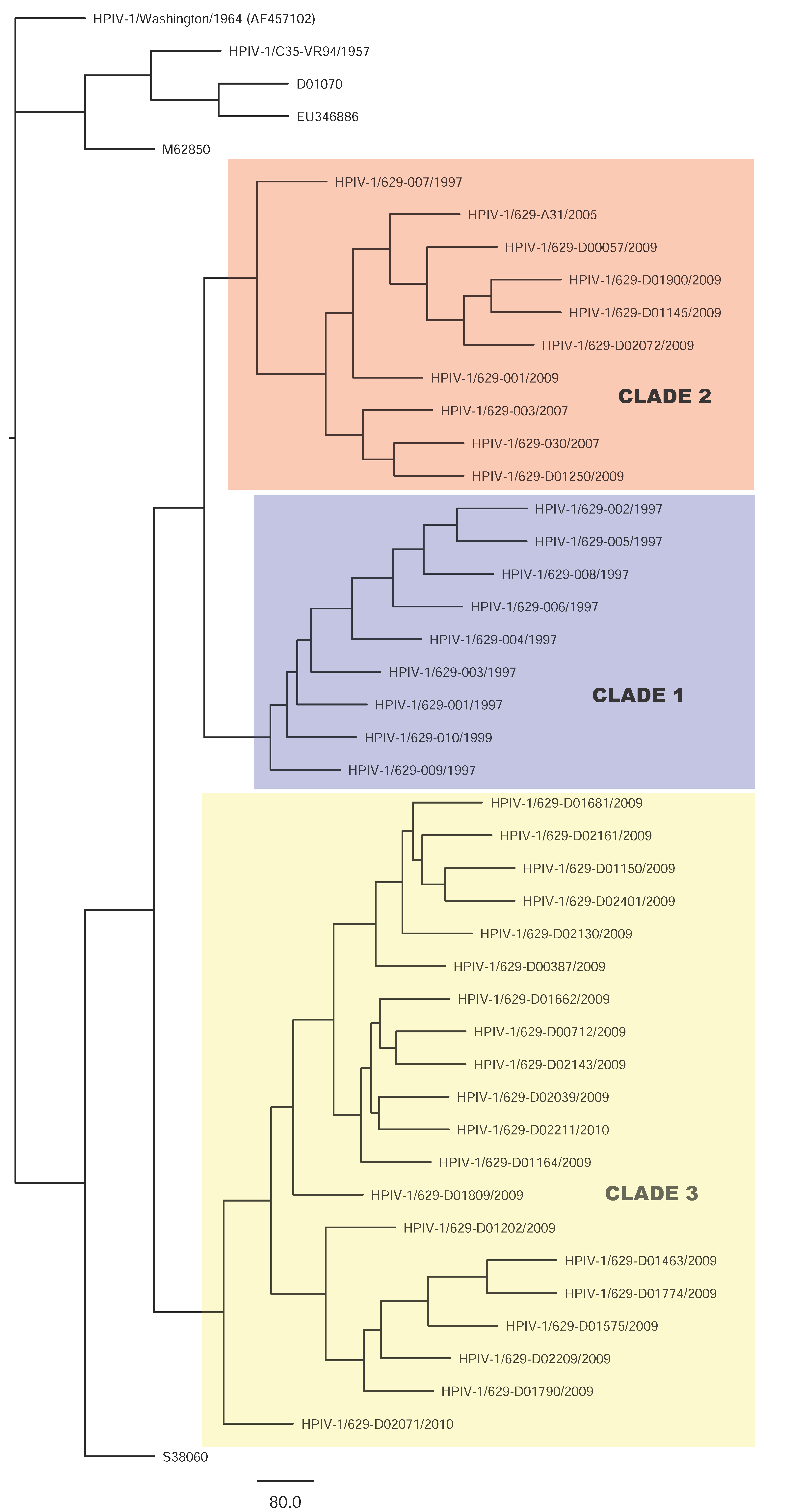

Supplement: Figure S2 — Phylogenetic relationship of HPIV-1 N gene including five sequences from GenBank with the maximum likelihood method in PAUP. The accession nos. of the five GenBank sequences are: D01070, EU346886, M62850, S38060 and AF457102. (TIFF) [file pone.0046048.s002.tiff]

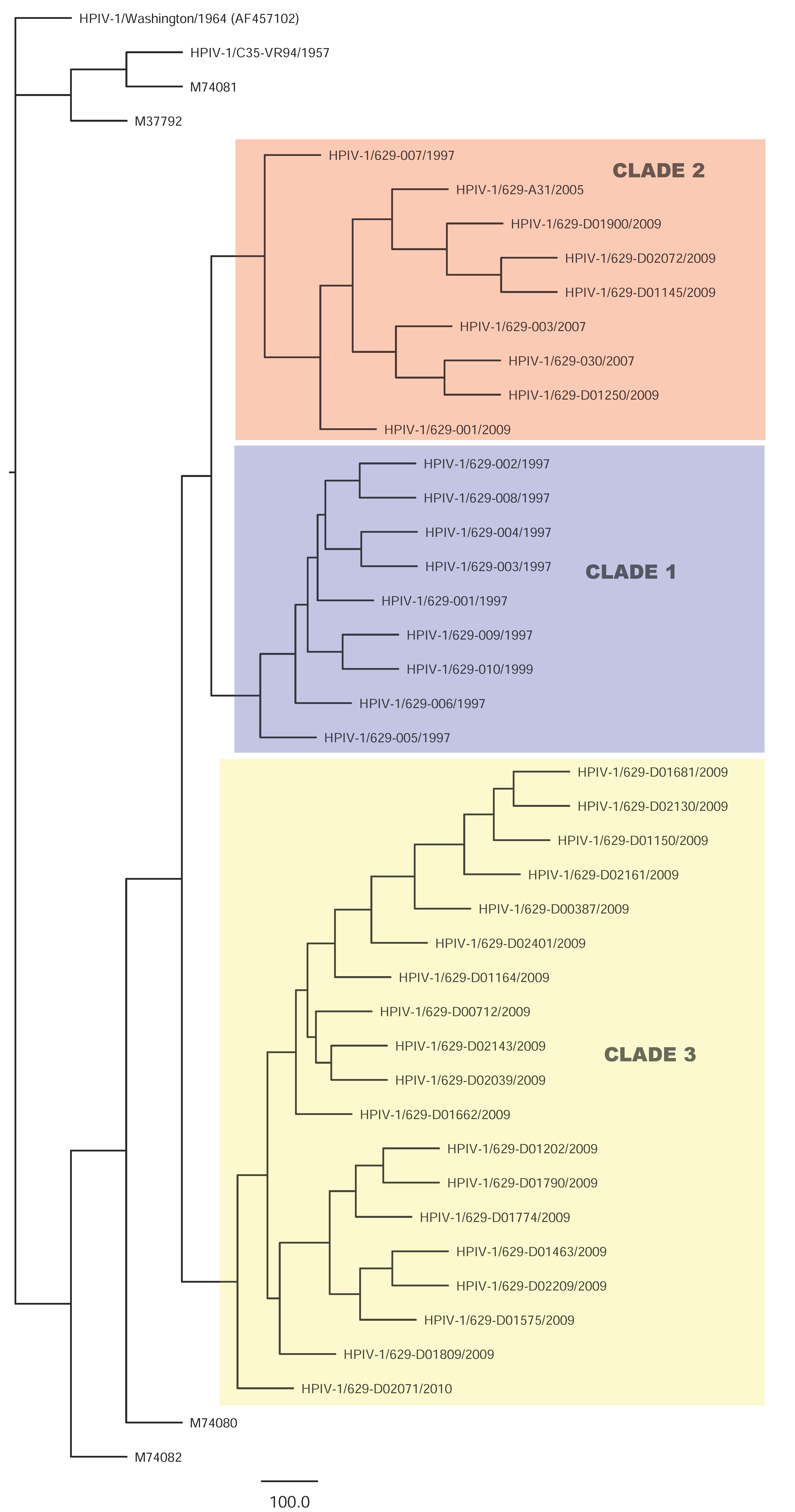

Supplement: Figure S3 — Phylogenetic relationship of HPIV-1 P gene including five sequences from GenBank with the maximum likelihood method in PAUP. The accession nos. of the five GenBank sequences are: M37792, M74080, M74081, M74082, and AF457102. (TIFF) [file pone.0046048.s003.tiff]

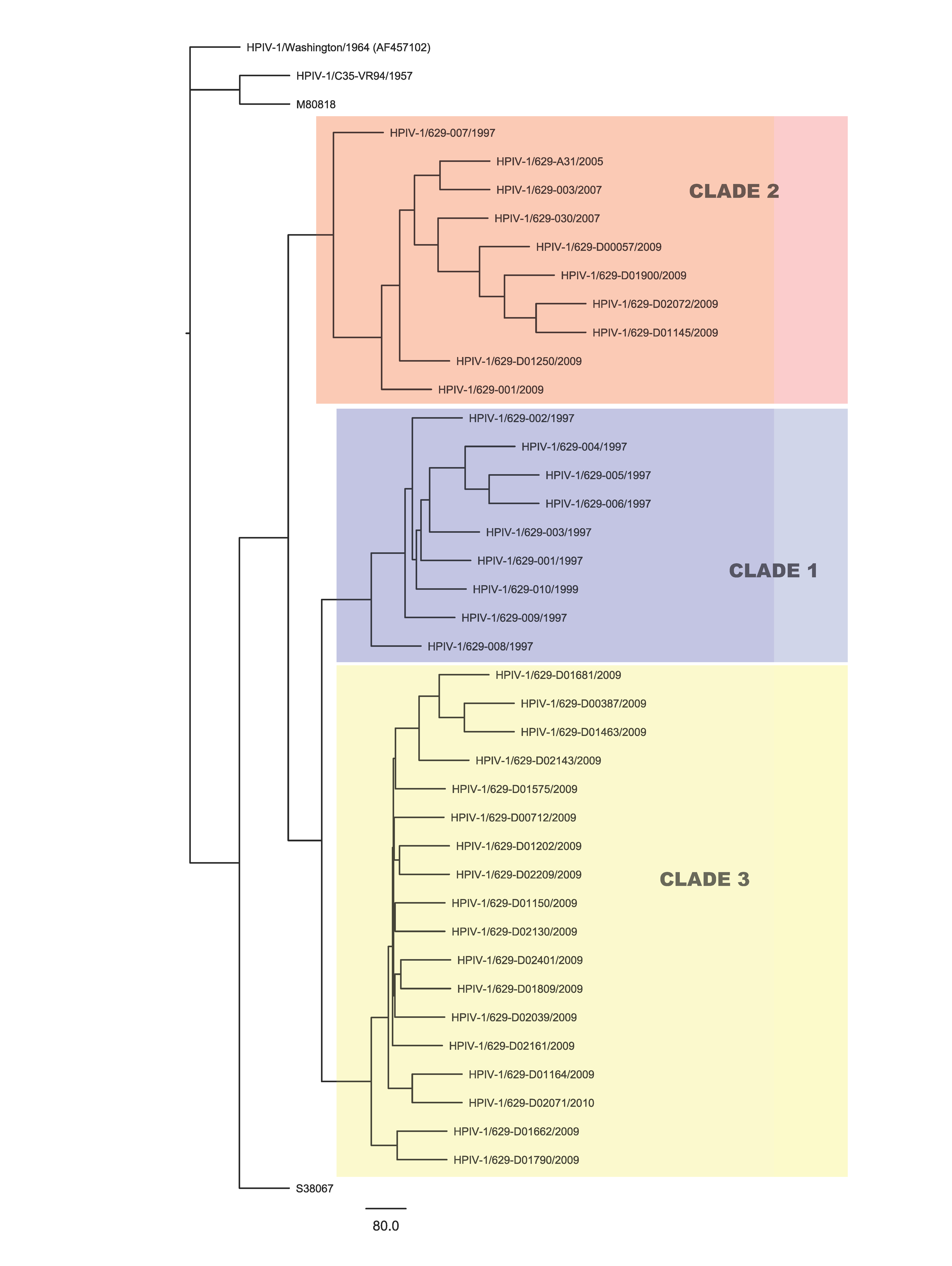

Supplement: Figure S4 — Phylogenetic relationship of HPIV-1 M gene including three sequences from GenBank with the maximum likelihood method in PAUP. The accession nos. of the three GenBank sequences are: M80818, S38067 and AF457102. (TIFF) [file pone.0046048.s004.tiff]

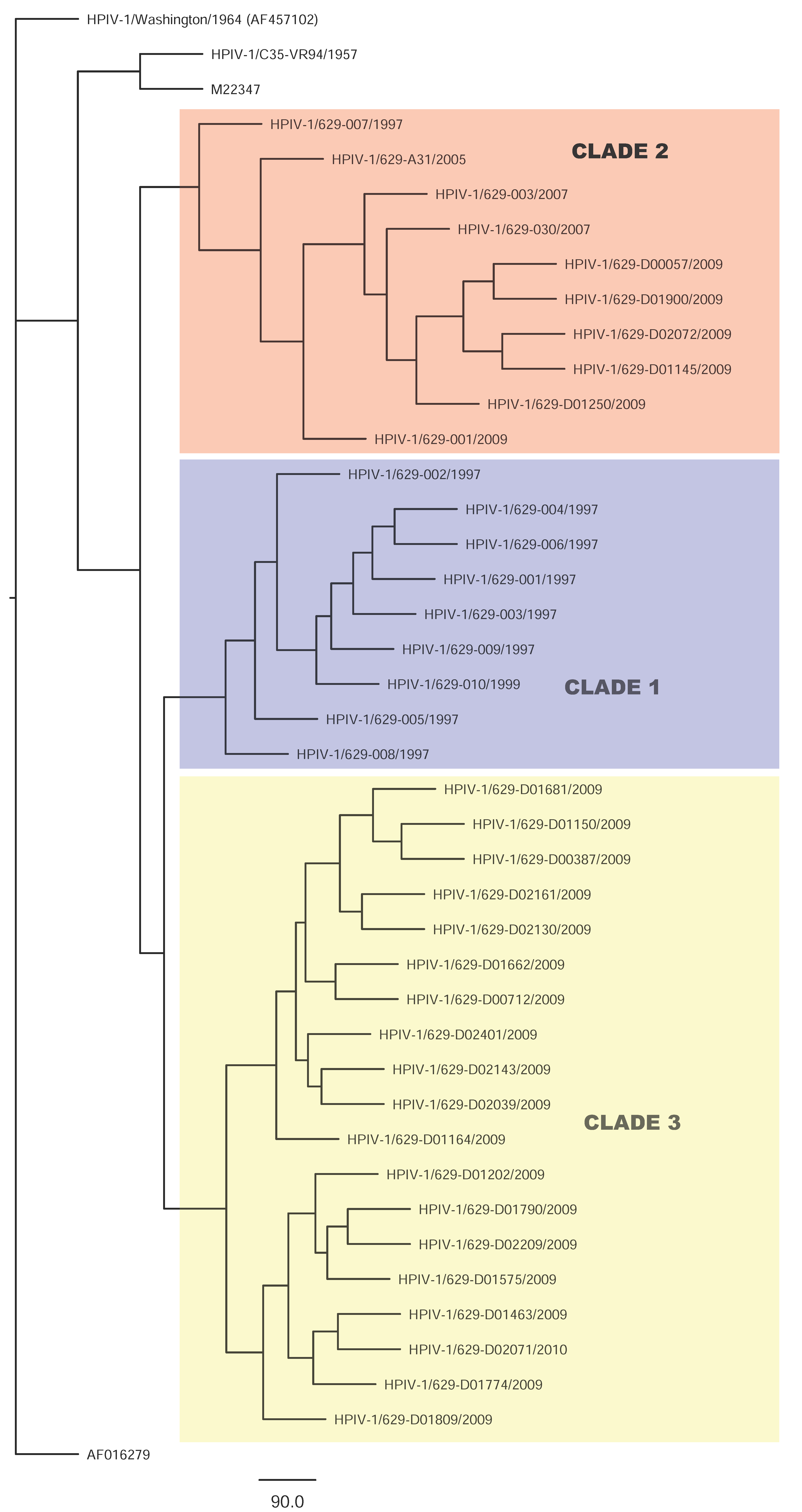

Supplement: Figure S5 — Phylogenetic relationship of HPIV-1 F gene including three sequences from GenBank with the maximum likelihood method in PAUP. The accession nos. of the three GenBank sequences are: AF016279, AF457102 and M22347. (TIFF) [file pone.0046048.s005.tiff]

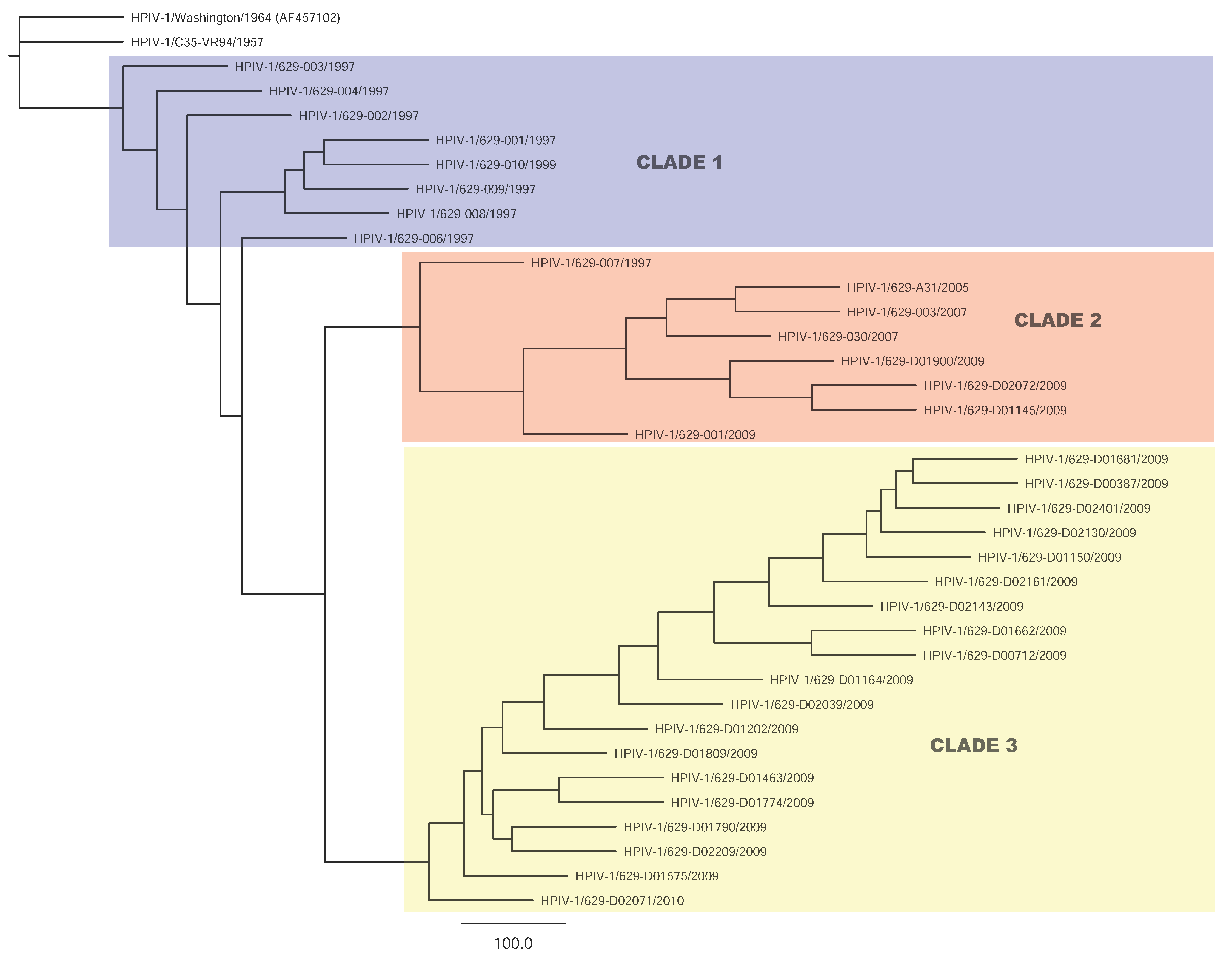

Supplement: Figure S6 — Phylogenetic relationship of HPIV-1 HN gene using sequences from the current study only with the maximum likelihood method in PAUP. (TIFF) [file pone.0046048.s006.tiff]

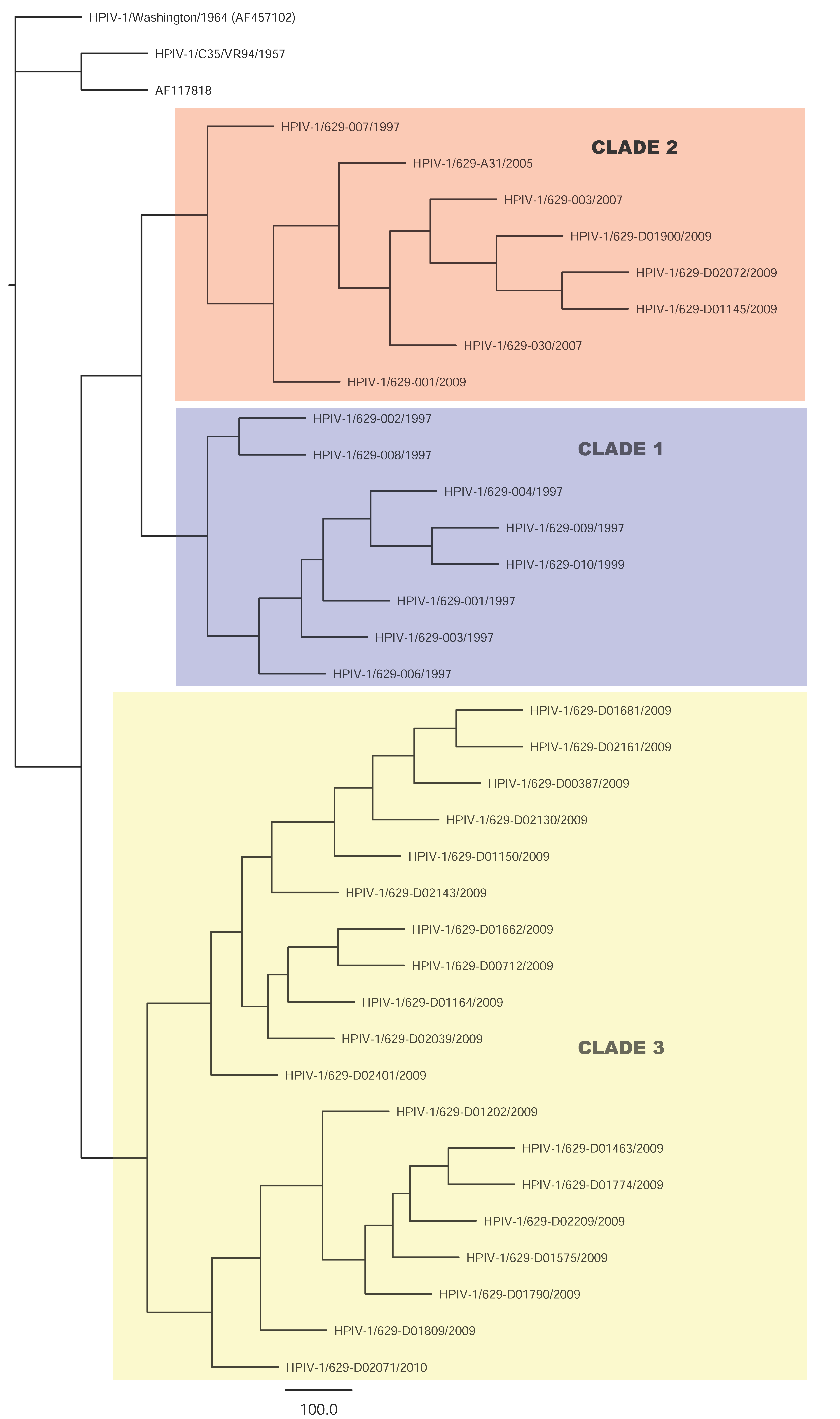

Supplement: Figure S8 — Phylogenetic relationship of HPIV-1 L gene including two sequences from GenBank with the maximum likelihood method in PAUP. The accession nos. of the two GenBank sequences are: AF117818 and AF457102. (TIFF) [file pone.0046048.s008.tiff]
